# Utility of ultrasound surveillance of fetal growth in patients with marginal or velamentous cord insertion

**DOI:** 10.1002/pmf2.70329

**Published:** 2026-05-29

**Authors:** Madeline Pence, Anna Madden‐Rusnak, Jermiah Crowder, Madeline Petrikas, Kobina Ghartey, Alison G. Cahill, Lorie M. Harper

**Affiliations:** ^1^ Department of Women's Health Dell Medical School at the University of Texas at Austin Austin Texas USA

**Keywords:** amniotic fluid index, antenatal testing, fetal growth, fetal growth restriction, marginal cord insertion, ultrasound, velamentous cord insertion

## Abstract

**Introduction:**

Abnormal cord insertion into the placenta (marginal and velamentous) has been associated with fetal growth restriction (FGR), leading some to recommend evaluation of fetal growth in the third trimester. The purpose of this study was to evaluate the timing of ultrasound surveillance in patients with marginal cord insertion (MCI) or velamentous cord insertion (VCI).

**Methods:**

Retrospective cohort of singleton pregnancies diagnosed with isolated MCI or VCI prior to 24 weeks at a single center from September 2021 to May 2025. Outcomes considered were diagnosis of FGR (estimated fetal weight [EFW] or abdominal circumference [AC] <10 percentile) and oligohydramnios (amniotic fluid index [AFI] ≤ 5 cm). The number of ultrasound scans needed to identify one abnormality (NNS) and 95% confidence interval (CI) were calculated for scans performed at 24+0–31+6 weeks and ≥ 32 weeks.

**Results:**

Among 248 patients with MCI meeting inclusion criteria, 710 ultrasounds were performed ≥ 24 weeks. A total of eight (3.2%) cases of FGR were diagnosed. Prior to 32 weeks, four cases of FGR were diagnosed (NNS 37.2, 95% CI, 14.9–135.8); however, half of these resolved on follow‐up ultrasound. After 32 weeks, the NNS to identify one case of FGR was 80.5 (95% CI, 31.7–294.6); FGR persisted on subsequent ultrasound(s) for all of these cases. Among 65 patients with VCI meeting inclusion criteria, 151 ultrasounds were performed. Two cases of FGR were diagnosed, both ≤ 32 weeks. The NNS to diagnose one case from 24+0–31+6 weeks was 26.5 (95% CI, 7.7–217.2). There were no cases of oligohydramnios diagnosed in either sample.

**Conclusion:**

For patients with MCI, half of FGR cases diagnosed ≤32 weeks resolved prior to delivery and were thus likely false‐positives, while all cases of FGR diagnosed ≥32 weeks persisted; thus, a single ultrasound at 32–36 weeks may be considered as a resource‐efficient monitoring approach. Among patients with VCI, both FGR cases (*n* = 2) were diagnosed at 30–32 weeks; initiation of growth ultrasounds after 28 weeks is recommended, although recommendations cannot be made regarding frequency based on our study.

## 1 INTRODUCTION

In singleton pregnancies, umbilical cord insertion abnormalities occur in approximately 6% of cases for marginal cord insertion (MCI) and 1.5% for velamentous cord insertion (VCI) [1]. MCI has been associated with adverse outcomes such as fetal growth restriction (FGR), low birthweight, and preterm delivery in some studies [2, 3]. Cord insertions located within 1 cm of the placental edge have been shown to carry a higher risk of FGR and low birthweight compared to insertions between 1.1 and 2.0 cm [3]. Neither the American College of Obstetricians and Gynecologists (ACOG) nor the Society for Maternal‐Fetal Medicine (SMFM) provides recommendations regarding third‐trimester ultrasound surveillance for pregnancies complicated by MCI; however, in clinical practice, serial ultrasound assessments are commonly performed.

In contrast, ACOG and SMFM recommend initiating antenatal fetal surveillance with fetal nonstress testing (NST) and/or biophysical profile (BPP) beginning at 36 weeks for pregnancies complicated by VCI due to its consistent association with adverse perinatal outcomes, including FGR, oligohydramnios, stillbirth, and low birthweight [4], with an odds ratio for stillbirth of 4.50 (95% confidence interval [CI], 2.18–9.27) [5]. Importantly, ACOG acknowledges that it remains unclear whether this elevated stillbirth risk is independent of or mediated through FGR, and the mechanistic pathway linking VCI to stillbirth has not been fully established. Regardless of mechanism, the absence of guidance on the optimal timing and frequency of monitoring prior to 36 weeks has led to considerable variation in clinical practice, and serial antenatal ultrasounds with fetal growth measurements are commonly performed, given the association between FGR and stillbirth [5].

The objective of this study was to evaluate the timing of ultrasound surveillance in patients with MCI or VCI. Specifically, we sought to determine the gestational age at which FGR and oligohydramnios are most frequently detected in order to inform evidence‐based strategies for antenatal monitoring and optimize surveillance practices while minimizing unnecessary interventions.

## 2 METHOD

We performed a retrospective cohort study of singleton pregnancies diagnosed with MCI or VCI at a single tertiary care center from September 2021 through May 2025. Institutional review board approval was obtained from Dell Medical School at The University of Texas at Austin (IRB #00001351).

Within the study period, we identified all patients seen at the Ascension Seton Medical Group (an affiliate of The University of Texas at Austin) antenatal testing unit who were diagnosed with MCI or VCI on ultrasound performed <24 weeks. In this study, MCI was defined as umbilical cord insertion ≤1.0 cm from the placental edge. Additional data were extracted from patient records, including ultrasound date, body mass index (BMI), medical diagnoses, pregnancy complications, estimated due date, fetal biometry measurements, and amniotic fluid index (AFI). Demographic information (race, ethnicity, insurance type, and marital status) was obtained through manual chart review by three trained study authors (J.P.C., M.B.P, and M.J.P.) following a standardized protocol and entered into a secure REDCap database [6]. During the study period, our institution's practice was to obtain at least one additional ultrasound after 28 weeks to assess fetal growth and amniotic fluid in patients with MCI or VCI.

Patients were included in the final analysis if they had a singleton pregnancy, were diagnosed with MCI or VCI on ultrasound <24 weeks, and had at least one additional ultrasound with fetal biometry and fluid measurements ≥24 weeks. Patients were excluded if they were diagnosed with FGR <24 weeks or if they had another indication for serial ultrasound scanning, including medical conditions (e.g., chronic hypertension, diabetes, BMI ≥ 35 kg/m^2^, maternal age ≤16 or ≥ 40 years at delivery) or pregnancy complications (e.g., fetal anomalies). For patients who developed gestational diabetes or pregnancy‐induced hypertension after diagnosis of MCI or VCI, all subsequent scans for that patient were excluded. Only the first pregnancy complicated by MCI or VCI was considered for patients with more than one pregnancy during the study period.

The primary outcomes were FGR (defined as estimated fetal weight [EFW] or abdominal circumference [AC] <10th percentile on the Hadlock fetal growth curve) [7] and oligohydramnios (AFI ≤5 cm) [8].

Patients who underwent one, two, or three or more ultrasound scans were compared using univariate statistical analyses. Continuous variables, including maternal age and BMI, were analyzed using ANOVA, and categorical variables, including race and parity, were analyzed using Fisher's exact test. Because of small sample sizes within individual non‐White racial categories, race was grouped into White and non‐White for statistical comparisons. A substantial proportion of patient‐reported variables, including race and ethnicity, were missing, and BMI was inconsistently documented in the medical record. Analyses were performed using available data for each variable.

If a patient underwent more than one ultrasound scan within a gestational age range, each scan was counted separately, but each patient contributed only one case of FGR or oligohydramnios (e.g., two scans with a single FGR diagnosis counted as two scans but one case). For patients diagnosed with FGR > 24 weeks, only scans prior to and on the day of diagnosis were included; subsequent scans were excluded because additional antenatal testing is indicated for FGR regardless of cord insertion type.

Two gestational age ranges were defined (24+0–31+6 weeks and 32+0–40+0 weeks) to represent roughly equal intervals from time of diagnosis to estimated time of delivery. For each gestational age range, we determined the total number of ultrasounds performed and the number of growth or fluid abnormalities identified. The number of ultrasounds needed to identify one abnormality (NNS) and the corresponding 95% CI were calculated for both gestational age ranges. The charts of patients who were diagnosed with FGR or oligohydramnios were reviewed to determine whether the diagnosis affected patient treatment. Analyses were completed in R Studio (Version 4.4.2) [9].

## 3 RESULTS

### 3.1 MCI

Of 633 patients with isolated MCI diagnosed during our 4‐year study period, 248 patients were included in the final analysis. The most common reasons for exclusion were BMI ≥ 35 kg/m^2^ (*n* = 100), diagnosis of MCI > 24 weeks (*n* = 90), and having only one ultrasound scan performed during the study period (*n* = 61). One patient was diagnosed with FGR < 4 weeks and one with oligohydramnios <24 weeks; both were excluded.

**FIGURE 1:**
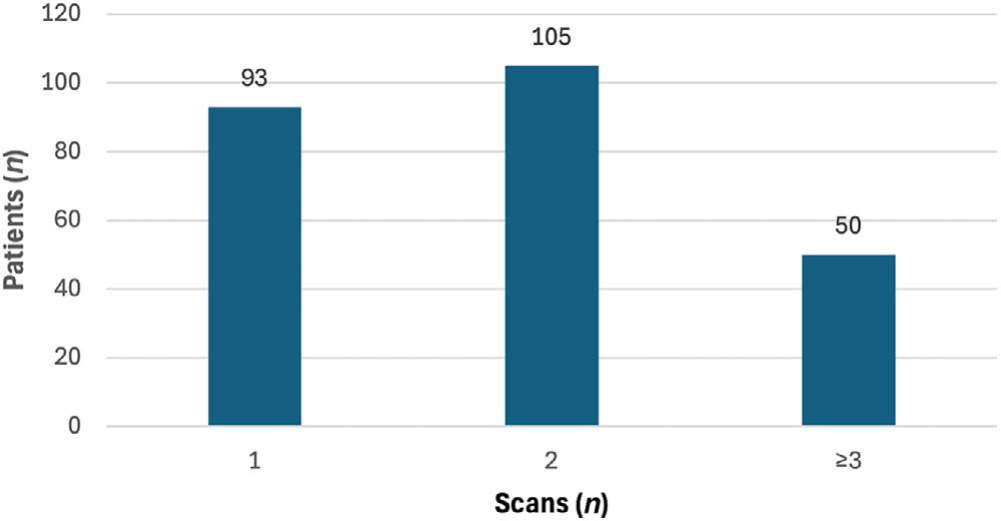
Number of scans performed among patients with marginal cord insertion.

The 248 patients included in the final analysis underwent a total of 710 scans with biometry and fluid measurements. Including initial ultrasound, 93 patients (37.5%) underwent two scans, 104 patients (41.9%) underwent three scans, and 51 patients (20.6%) underwent four or more scans (Figure 1). Patients who had two, three, and four or more scans were statistically similar with respect to race, ethnicity, primary language, marital status, insurance status, parity, and BMI (all *p* > 0.05, Table 1).

**TABLE 1:** Maternal and pregnancy characteristics by number of ultrasounds performed after diagnosis of marginal cord insertion.

	Number of ultrasound scans						
Variable	2 (*n* = 93)	% missing	3 (*n* = 105)	% missing	≥4 (*n* = 50)	% missing	*p* value
Maternal age, mean ± SD	30.4 ± 5.1	0.0	29.8 ± 4.9	0.0	31.0 ± 5.8	0.0	0.27
Maternal race, *n*	12.9		16.3		15.7		0.80
White	54 (58.1%)		63 (60.0%)		32 (64.0%)		
Non‐White	39 (41.9%)		42 (40.0%)		18 (36.0%)		
Black	9 (9.7%)		7 (6.7%)		4 (8.0%)		
Asian	8 (8.6%)		9 (8.6%)		5 (10.0%)		
Native Hawaiian or other Pacific Islander	0 (0.0)		1 (1.0%)		0 (0.0)		
Mexican American Indian	0 (0.0)		0 (0.0)		1 (2.0%)		
Other	10 (10.7%)		25 (7.6%)		0 (0.0)		
Ethnicity, *n*	3.2		3.8		6.0		0.13
Non‐Hispanic/Latina	50 (53.8%)		52 (49.5%)		34 (68.0%)		
Hispanic/Latina	40 (43.0%)		49 (46.7%)		13 (26.0%)		
Primary language, *n*	0.0		1.0		2.0		0.36
English	74 (79.6%)		77 (73.3%)		43 (86.0%)		
Spanish	16 (17.2%)		26 (24.8%)		6 (12.0%)		
Other	3 (3.2%)		2 (1.9%)		1 (2.0%)		
Marital Status, *n*	2.2		0		2.0		0.30
Married	73 (78.5%)		75 (71.4%)		38 (76.0%		
Single	18 (19.4%)		27 (25.7%)		11 (22.0%)		
Divorced/separated	0		3 (2.9%)		1 (2.0%)		
Insurance, *n*	0.0		0.0		0.0		0.91
Private	62 (66.7%)		69 (65.7%)		35 (70.0%)		
Public	29 (31.2%)		32 (30.5%)		13 (26.0%)		
Self‐pay	2 (2.2%)		4 (3.8%)		2 (4.0%)		
Parity, *n*	0.0		0.0		0.0		0.16
Nulliparous	54 (58.7%)		55 (52.4%)		21 (42.0%)		
Multiparous	38 (41.3%)		50 (47.6%)		29 (58.0%)		
BMI, kg/m^2^ ^a^	25.8 ± 3.8	20.4	25.4 ± 3.3	13.5	24.7 ± 3.1	13.7	0.47

Abbreviation: BMI, body mass index.

^a^ Data shown as mean ± SD.

FGR was diagnosed in eight pregnancies (3.2%, Table 2). Of these, four (1.6%) were diagnosed between 24+0–31+6 weeks and four (1.6%) were diagnosed between 32 and 40 weeks. The number of ultrasounds needed to be performed to detect one case of FGR (NNS) increased from 37.2 (95% CI, 14.9–135.8) at 24+0–31+6 weeks to 80.5 (95% CI, 31.7–294.6) at 32–40 weeks. Two (50%) of the four cases diagnosed between 24 weeks and 31+6 weeks resolved prior to delivery (Table 3). No cases of FGR resolved prior to delivery when diagnosed after 32 weeks. There were no cases of oligohydramnios diagnosed in the sample.

**TABLE 2:** Test characteristics of ultrasound scans by gestational age among patients with MCI.

Gestational age in weeks	US scans performed, *n*	FGR cases diagnosed on US, *n*	Oligohydramnios cases diagnosed on US, *n*	NNS (95% CI)
24+0–31+6 weeks	149	4^a^	0	37.2 (14.9–135.8)
32+0–40+0 weeks	322	4	0	80.5 (31.7–294.6)

Abbreviations: CI, confidence interval; FGR, fetal growth restriction; MCI, marginal cord insertion; NNS, number needed to screen; US, ultrasound.

^a^ Two out of four FGR diagnoses between 24 and 31.6 weeks’ gestations resolved prior to delivery.

**TABLE 3:** Growth parameters and resolution of fetal growth restriction (FGR) among patients with marginal cord insertion.

Case	Gestational age at diagnosis	Fetal growth percentile at time of diagnosis of FGR (%)	FGR resolved on subsequent scan?
1	26+6 weeks	<1	No
2	29+1 weeks	5	Yes, at 32+3 weeks
3	29+6 weeks	7	Yes, at 33+6 weeks
4	31+0 weeks	<1	No
5	32+0 weeks	6	No
6	32+4 weeks	2	N/a, subsequent scan not performed
7	33+1 weeks	3	No
8	39+5 weeks	<1	N/a, subsequent scan not performed

### 3.2 VCI

Of 168 patients with isolated VCI diagnosed during our 4‐year study period, 65 patients were included. The most common reasons for exclusion were BMI ≥ 35 kg/m^2^ (*n* = 29), age ≥ 40 years (*n* = 22), and diagnosis of VCI > 24 weeks (*n* = 19). Four patients were diagnosed with FGR < 24 weeks and were excluded from analysis.

**FIGURE 2:**
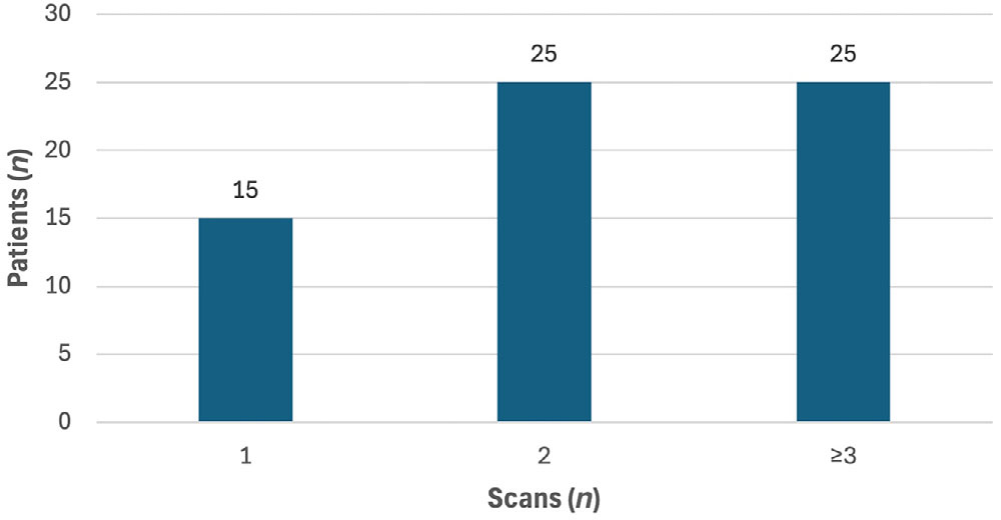
Number of scans performed among patients with velamentous cord insertion.

The 65 patients included in the final analysis underwent 151 total scans with biometry and fluid measurements. Including the initial anatomy scan, 15 patients (23.1%) underwent two scans, 25 patients (38.5%) underwent three scans, and 25 patients (38.5%) underwent four or more scans (Figure 2). Patients who had two, three, and four or more scans were similar with respect to ethnicity, primary language, marital status, insurance status, parity, and BMI (Table 4). Patients who underwent two versus three versus four or more scans were significantly different with respect to race (*p *= 0.03), with more non‐White than White patients in the group with at least four scans, though this and other post hoc pairwise comparisons between scan groups were not statistically significant following Bonferroni adjustments (*p* > 0.05).

**TABLE 4:** Maternal and pregnancy characteristics by number of ultrasounds performed after diagnosis of VCI.

	Number of ultrasound scans						
Variable	2 (*n* = 15)	% missing	3 (*n* = 25)	% missing	≥4 (*n* = 25)	% missing	*p* value
Maternal age, mean ± SD	29.9 ± 6.4	0	30.9 ± 5.0	0	31.1 ± 5.2	0	0.79
Maternal race, *n*	33.3		28.0		8.0		0.03
White	9 (60.0%)		8 (32.0%)		17 (68.0%)		
Non‐White	6 (40.0%)		17 (68.0%)		8 (32.0%)		
Black	0		3 (12.0%)		0		
Asian	1 (6.7%)		0		4 (16.0%)		
American Indian or Alaska Native	0		1 (4.0%)		1 (4.0%)		
Mexican	0		1 (4.0%)		0		
Other/unknown	5 (33.3%)		12 (48.0%)		4 (16.0%)		
Ethnicity, *n*	0		4.0		4.0		0.50
Non‐Hispanic/Latina	7 (46.7%)		7 (28.0%)		12 (48.0%)		
Hispanic/Latina	8 (53.3%)		17 (68.0%)		12 (48.0%)		
Primary language, *n*	6.7		0		0		0.08
English	9 (60.0%)		15 (60.0%)		21 (84.0%)		
Spanish	5 (33.3%)		10 (40.0%)		3 (12.0%)		
Other	1 (6.7%)		0		1 (4.0%)		
Marital Status, *n*	0		0		0		0.56
Married	12 (80.0%)		16 (64.0%)		19 (76.0%)		
Single	3 (20.0%)		9 (36.0%)		6 (24.0%)		
Insurance, *n*	0		0				0.39
Private	9 (60.0%)		13 (52.0%)		16 (64.0%)		
Public	6 (40.0%)		12 (48.0%)		7 (28.0%)		
Self‐pay	0		0		2 (8.0%)		
Parity, *n*	0		0				0.26
Nulliparous	9 (64.3%)		10 (40.0%)		15 (60.0%)		
Multiparous	5 (35.7%)		15 (60.0%)		10 (40.0%)		
BMI, kg/m^2^ ^a^	24.8 ± 4.1	20.0	26.9 ± 3.8	48.0	25.9 ± 3.3	40.0	0.29

Abbreviations: BMI, body mass index; VCI, velamentous cord insertion.

^a^ Data shown as mean ± SD.

FGR was diagnosed in two pregnancies with VCI (3.1%, Table 5). Both cases were diagnosed between 24 weeks and 31+6 weeks at an average of 30+3 weeks. The NNS at this gestational age range was 26.5 (95% CI, 7.7–217.2). There were no additional cases of FGR diagnosed between 32 and 40 weeks. No cases of oligohydramnios were diagnosed in either gestational age range.

**TABLE 5:** Test characteristics of ultrasound scans at each gestational age among patients with VCI.

Gestational age in weeks	US scans performed, *n*	FGR cases diagnosed on US, *n*	Oligohydramnios cases diagnosed on US, *n*	NNS^a^ (95% CI)
24+0–31+6 weeks	53	2	0	26.5 (7.7–217.2)
32+0–40+0 weeks	98	0	0	–

Abbreviations: FGR, fetal growth restriction; NNS, number needed to screen; US, ultrasound; VCI, velamentous cord insertion.

^a^ Reflects a combination of FGR and oligohydramnios.

## 4 DISCUSSION

In our cohort of patients with umbilical cord insertion abnormalities without comorbidities, few cases of FGR were diagnosed by antenatal ultrasound. The number of ultrasounds needed to be performed to detect one case of FGR was high at both gestational age ranges studied. Notably, half of the cases of early‐onset FGR in patients with isolated MCI resolved by the time of delivery, indicating a high false‐positive rate for scans performed at an early gestational age in this population. These findings have practical implications for ultrasound surveillance protocols. Notably, both false‐positive cases of early FGR had growth percentiles of 5%–7% at diagnosis, whereas both persistent early cases of FGR were severe, with growth percentiles of <1%. Given this, along with the persistence of all cases identified after 32 weeks, a growth ultrasound at 32–36 weeks may represent a resource‐efficient approach to surveillance for most patients, although clinicians should remain attentive to severe early FGR, which may warrant follow up regardless of protocol. Prospective studies with larger sample sizes are needed to determine whether early percentile thresholds can reliably distinguish true from false‐positive cases of FGR in this population.

Among patients with isolated VCI, only two cases of FGR were identified, both between 24 weeks and 31+6 weeks; 27 ultrasound scans needed to be performed to detect one case in this gestational age range. Among patients with VCI, there were no new cases of FGR diagnosed after 32 weeks, suggesting growth should be evaluated at or after 28 weeks. However, given the small number of cases and wide CIs around our estimates, we are unable to make an evidence‐based recommendation on the frequency of ultrasound surveillance in this population. Larger prospective studies are needed before any practice‐changing recommendations can be made.

There is strong evidence that VCI is associated with stillbirth, with an estimated odds ratio of 4.50 (95% CI, 2.18–9.27) [4]. VCI has also been associated with adverse pregnancy outcomes, including small‐for‐gestational‐age (SGA) at birth, preterm delivery, and emergency cesarean section [1]. Although ACOG acknowledges that it is unclear whether the increased risk of stillbirth in this population is independent of FGR, they recommend weekly antenatal testing with NST or BPP beginning at 36 weeks for those with VCI [4]. While some investigators have advocated for early detection and surveillance throughout pregnancy [2], there are currently no evidence‐based risk‐reducing interventions or standardized management pathways for pregnancies complicated by VCI [10].

In contrast, the association between MCI and adverse pregnancy outcomes, including FGR, is less well‐established. Neither ACOG nor SMFM recommends additional antenatal surveillance for pregnancies complicated by MCI, although several studies have investigated pregnancy outcomes in this population. In a large retrospective cohort study of 1819 singleton gestations, Asoglu et al. [11] found that MCI was not associated with adverse pregnancy outcomes and concluded that increased antepartum surveillance may not be necessary. Conversely, a systematic review and meta‐analysis of 15 studies found an association between MCI and increased risk of SGA (RR, 1.25; 95% CI, 1.21–1.29) and stillbirth (RR, 1.97; 95% CI 1.02–3.78) [12]. Heterogeneity in findings among studies may reflect differences in how MCI is defined based on the distance of the cord insertion from the placental edge, as cord insertion ≤ 1.0 cm from the placental margin has been more strongly associated with adverse outcomes than insertion 1.1–2.0 cm from the placental edge [3]. Despite the absence of formal recommendations for antenatal testing among patients with MCI, many institutions begin surveillance ultrasounds as early as 24 weeks for this population. These practices contribute to considerable healthcare utilization and patient burden with uncertain clinical benefit.

One notable finding of this study was the overall low incidence of FGR within our cohort. Only eight pregnancies (3.2%) with MCI and two pregnancies (3.1%) with VCI were diagnosed with FGR. This rate is much lower than would be expected based on the Hadlock fetal growth charts [7]. Several factors may explain this discrepancy. The Hadlock growth curve was derived from a predominantly White, middle‐class, low‐risk population in the 1980s and 1990s, which likely differs from our study population which included a higher proportion of Hispanic and publicly insured patients. Additionally, our stringent exclusion criteria were designed to isolate the effect of abnormal cord insertion on fetal growth. While this served to minimize confounding from other medical or pregnancy‐related comorbidities, it may have created a group that was at lower risk of FGR at baseline. The exclusion of a significant number of patients from the analysis may limit the generalizability of our findings.

To our knowledge, our study is the first to examine the optimal timing and frequency of ultrasound surveillance for placental cord insertion abnormalities. A strength of our study is the inclusion only of patients with initial ultrasound prior to 24 weeks, which reduces the likelihood of inaccurate dating as an artificial contributor to FGR classification. We also excluded patients with other indications for ultrasound surveillance, including pre‐existing medical conditions and pregnancy complications after diagnosis, such that the ultrasound findings and FGR diagnoses observed in our cohort could be more confidently attributed to the cord insertion abnormality itself rather than to competing maternal or fetal conditions.

Interestingly, despite the application of a more restrictive and likely clinically significant definition of MCI as cord insertion ≤ 1.0 cm from the placental margin, our cohort still exhibited a low overall incidence of FGR, with a 50% false‐positive rate among those with an early diagnosis of FGR. Of note, our use of this more restrictive definition limits direct comparability with existing literature that utilizes a less restrictive definition of cord insertion as ≤ 2.0 cm from the placental margin.

Several limitations of this study warrant consideration. The small sample sizes in both cohorts, and particularly the low prevalence of isolated VCI, limit statistical precision, result in wide CIs, and preclude definitive recommendations regarding optimal surveillance timing; findings should be considered hypothesis‐generating rather than practice‐changing. The main reason for our small sample size was our strict inclusion criteria to isolate MCI and VCI as the sole indications for ultrasound, prioritizing internal validity and clinical interpretability. By first defining the relationship in a low‐confounding cohort, future studies are better positioned to extend this work to more complex populations, incorporate comorbid conditions, and evaluate implementation within routine clinical care. Our study used FGR as a surrogate marker for perinatal morbidity and did not address stillbirth, which is the primary driver of current surveillance recommendations for VCI and may occur independently of FGR. This limits direct assessment of the impact of ultrasound surveillance on perinatal outcomes. Although no new cases of FGR were diagnosed after 32 weeks, and a binomial upper‐confidence‐limit approximation suggests that the true risk of incident FGR after 32 weeks is unlikely to exceed approximately 5%, this estimate remains limited by sample size. Comparisons across patient subgroups were limited to univariate analyses; patients who underwent more ultrasound scans may differ systematically from those who received fewer in ways not captured by available data, and the absence of multivariable adjustment for potential confounders limits the validity of these comparisons. Although there were substantial missing data for race, ethnicity, and BMI, limiting demographic comparisons, these variables are not expected to affect the diagnostic performance of ultrasound for FGR and thus are unlikely to bias the primary outcome. Finally, the single‐center, retrospective design limits generalizability and introduces selection bias. Specifically, there was notable variation in surveillance practices, as evidenced by the variation in the number of ultrasounds performed in our population. This was actually the catalyst of the study; little evidence exists to inform the timing and frequency of ultrasounds in the setting of MCI and VCI, which leads to significant provider variation. This study provides an initial step in clarifying and optimizing ultrasound use in these cases, although prospective evaluation of a large cohort of isolated MCI and VCI would further elucidate the optimal timing of ultrasound.

## 5 CONCLUSION

Umbilical cord insertion abnormalities are common, and antenatal screening of pregnancies complicated only by abnormal cord insertion is resource‐intensive and demanding on the patient. Further data are needed to balance the demands on patients and clinics with the desire for close monitoring of FGR to prevent stillbirth. In this cohort, there was a low incidence of FGR and a relatively large number of scans needed to be performed to identify one case of FGR. Among patients with MCI without significant comorbidities, half of the cases of FGR diagnosed ≤ 32 weeks resolved prior to delivery and were thus likely false‐positives. All cases of FGR diagnosed ≥ 32 weeks persisted until delivery. Therefore, if an ultrasound surveillance screening protocol for patients with MCI is used, a single ultrasound at 32–36 weeks may be considered as a resource‐efficient approach to monitoring. Among patients with isolated VCI, few cases of FGR were diagnosed, and all were diagnosed at 30–32 weeks. However, given the small number of cases, this observation should be interpreted with caution, as larger prospective studies are needed before any practice‐changing recommendations can be made. In this study, for both the MCI and VCI cohorts, wide CIs, retrospective design, and lack of stillbirth data preclude definitive recommendations on ultrasound surveillance timing.

## CONFLICT OF INTEREST STATEMENT

The authors declare no conflicts of interest.

## FUNDING INFORMATION

The authors received no specific funding for this work.

## Data Availability

The data that support the findings of this study are available on request from the corresponding author. The data are not publicly available due to privacy or ethical restrictions.
